# Peak ground acceleration prediction for on-site earthquake early warning with deep learning

**DOI:** 10.1038/s41598-024-56004-6

**Published:** 2024-03-06

**Authors:** Yanqiong Liu, Qingxu Zhao, Yanwei Wang

**Affiliations:** 1 China Earthquake Network Center, Beijing, China; 2 Key Laboratory of Urban Security and Disaster Engineering of China Ministry of Education, Beijing University of Technology, Beijing, China; 3 Guangxi Key Laboratory of Geomechanics and Geotechnical Engineering, Guilin University of Technology, Guilin, China

**Keywords:** On-site earthquake early warning, Ground motion, Peak ground acceleration, Deep learning, Convolution neural network, Seismology, Natural hazards

## Abstract

Rapid and accurate prediction of peak ground acceleration (PGA) is an important basis for determining seismic damage through on-site earthquake early warning (EEW). The current on-site EEW uses the feature parameters of the first arrival P-wave to predict PGA, but the selection of these feature parameters is limited by human experience, which limits the accuracy and timeliness of predicting peak ground acceleration (PGA). Therefore, an end-to-end deep learning model is proposed for predicting PGA (DLPGA) based on convolutional neural networks (CNNs). In DLPGA, the vertical initial arrival 3–6 s seismic wave from a single station is used as input, and PGA is used as output. Features are automatically extracted through a multilayer CNN to achieve rapid PGA prediction. The DLPGA is trained, verified, and tested using Japanese seismic records. It is shown that compared to the widely used peak displacement (Pd) method, the correlation coefficient of DLPGA for predicting PGA has increased by 12–23%, the standard deviation of error has decreased by 22–25%, and the error mean has decreased by 6.92–19.66% with the initial 3–6 s seismic waves. In particular, the accuracy of DLPGA for predicting PGA with the initial 3 s seismic wave is better than that of Pd for predicting PGA with the initial 6 s seismic wave. In addition, using the generalization test of Chilean seismic records, it is found that DLPGA has better generalization ability than Pd, and the accuracy of distinguishing ground motion destructiveness is improved by 35–150%. These results confirm that DLPGA has significant accuracy and timeliness advantages over artificially defined feature parameters in predicting PGA, which can greatly improve the effect of on-site EEW in judging the destructiveness of ground motion.

## Introduction

The Earthquake Early Warning (EEW) system is a seismic engineering system that plays an important role in earthquake emergency response. After an earthquake occurs, the EEW system can send an alarm within seconds to tens of seconds before the destructive seismic wave reaches the target area, allowing users in the target area to escape in time. At present, the EEW system has been established or is being established in countries with more active earthquakes in the world^1,2^, such as Japan^3,4^, USA^5^, China^6^, Mexico^7^, Italy^8^, and India^9^. Moreover, the EEW system has successfully warned against multiple earthquakes and played a crucial role in disaster reduction^10–13^. The key to the success or failure of the EEW system is whether it can quickly and accurately estimate the degree of damage to the target area. This is not only the basis for the EEW system to issue warning information but also the basis for users to take emergency measures.

Peak ground acceleration (PGA) is an important parameter used in EEW systems to estimate the damage level of the target area. PGA directly reflects the intensity of ground motion and has a good correlation with intensity and disaster^2,14,15^, which is widely used to determine seismic damage, such as earthquake disaster assessment^16^, probabilistic seismic disaster analysis (PSHA)^17^, and earthquake warning for high-speed rail^18^. Unlike conventional seismic monitoring, EEW systems need to predict the PGA before it is observed. After predicting PGA in EEW systems, PGA can be used to calculate intensity or compare thresholds to estimate the degree of earthquake damage^19–24^. According to the different implementation methods, EEW systems can be divided into regional EEW and on-site EEW. Regional EEW monitors the initial seismic waves at multiple stations to estimate the earthquake magnitude and epicenter location and then further predicts the seismic intensity and damage level of the target area. Onsite EEW predicts the seismic intensity and damage level at the location of a single station based only on the first arrival seismic wave monitored at that station, without determining the earthquake magnitude and epicenter location. The two EEW systems predict PGA in different ways. Regional EEW systems predict PGA by using ground motion prediction equations (GMPEs)^25–30^ or machine learning prediction models^31–33^ after determining seismic source parameters such as magnitude and epicenter location. The disadvantage of this method lies in that the accuracy of the prediction results depends on the accuracy of the magnitude, epicenter location, and GMPEs or machine learning models. In addition, because the magnitude and epicenter location generally need to wait for multiple stations to trigger (more than three) to be determined, the timeliness of predicting seismic parameters is limited^34,35^. The on-site EEW system predicts PGA based on the first arrival seismic wave at a single station. Compared with the regional EEW system, this method simplifies the calculation process because it does not require magnitude and epicenter location and has higher timeliness than the regional EEW method. However, the drawback of the on-site EEW system is the limited available information (only the initial few seconds of seismic waves from a single station), which makes it difficult to ensure the accuracy of predicting PGA and may lead to false alarms or missed alarms. Improving the accuracy of on-site EEW for predicting PGA has become an important research topic in the field of earthquake warning in recent years.

The key to predicting PGA in on-site EEW is to find characteristic parameters related to PGA from the first arrival seismic waves. Characteristic parameters such as peak displacement (Pd), peak velocity, peak acceleration, and effective dominant period of first arrival seismic waves are widely used for predicting PGA. Hsu et al.^36^ inputted the characteristic parameters of multiple first-arrival seismic waves into support vector regression (SVR) to predict PGA, which showed good results for multiple earthquakes^12,22,24^. To consider the impact of site conditions on predicting PGA, Hsu et al.^37^ further proposed a method for predicting PGA by inputting 19 characteristic parameters into a neural network. In addition, Wang et al.^38^ used 8 typical characteristic parameters of first arrival seismic waves as inputs to the long short-term memory neural network (LSTM), and the accuracy of predicting PGA was better than that of the peak displacement (Pd) method. However, these input characteristic parameters are all artificially defined, which cannot avoid human subjectivity and can only be related to PGA in certain aspects of the first arrival seismic wave, thereby affecting the accuracy of prediction. Compared to manually extracting features from first arrival seismic waves, deep learning methods can overcome human subjectivity and automatically extract more comprehensive features from first arrival waves. Deep learning is currently the most cutting-edge and popular type of machine learning algorithm and has achieved great success in fields such as speech recognition, image recognition, and translation^39^. In particular, in recent years, deep learning represented by convolutional neural networks (CNNs) has achieved remarkable results in many aspects of seismic engineering. Scholars have used CNNs to automatically extract features from first-arrival seismic waves for phase picking^40–42^, magnitude estimation^43–45^, earthquake positioning^40,46^, earthquake disaster assessment^47,48^, and prediction of ground motion parameters^49^, among other aspects. In particular, in the past 2 years, deep learning has also been used to predict PGA. Jozinović et al.^50^ used seismic data from central Italy and successfully predicted PGA using the initial 7–15 s three-component seismic waves from multiple stations as inputs to CNN, indicating that this method has similar errors to the GMPE method developed by Bindi et al.^26^. To fully utilize the information of initial P-waves, Hsu et al.^51^ proposed a method for predicting PGA by inputting P-waves into a CNN after multiscale and multidomain preprocessing and showed that the accuracy of this method exceeded that of the SVR method proposed in 2013^36^. Chiang et al.^52^ inputted three-component first-arrival seismic waves into a CNN to predict whether the PGA exceeded the threshold in a classified form. From previous research, it can be seen that deep learning methods can automatically extract features related to PGA from first arrival seismic waves, and the accuracy of predicting PGA is much better than that based on manually defined characteristic parameters. However, the shortcomings of the methods proposed in these studies are that they require a longer first arrival seismic wave input and cannot meet the timeliness requirements of the onsite EEW system (usually starting to predict PGA at the first 3 s P wave)^53–55^. The complex manual preprocessing of the input first arrival seismic waves not only fails to overcome the subjective influence of humans but also increases the complexity of algorithm implementation. Therefore, it is necessary to develop a deep learning model that meets the timeliness requirements of earthquake warning and can avoid human interference to improve the prediction effect of PGA and improve the accuracy of the EEW system in identifying earthquake damage.

Herein, we propose a deep learning model (DLPGA) for predicting PGA in on-site EEW based on a multilayer CNN. DLPGA achieves PGA prediction in an end-to-end form by automatically extracting features from a single station's initial 3–6 s vertical seismic wave. First, a training dataset, validation dataset, and testing dataset were established using Japan's 31,300 sets of three-component surface acceleration records. A CNN model was designed, and its predictive performance was tested. Then, to evaluate the predictive performance of the trained CNN model in different regions, a generalization ability test was conducted using Chile's 5053 sets of three-component acceleration records. The results show that the proposed DLPGA has a significantly better performance in predicting PGA than the commonly used Pd method, making on-site EEW more accurate in identifying seismic damage.

## Datasets

**Figure 1:**
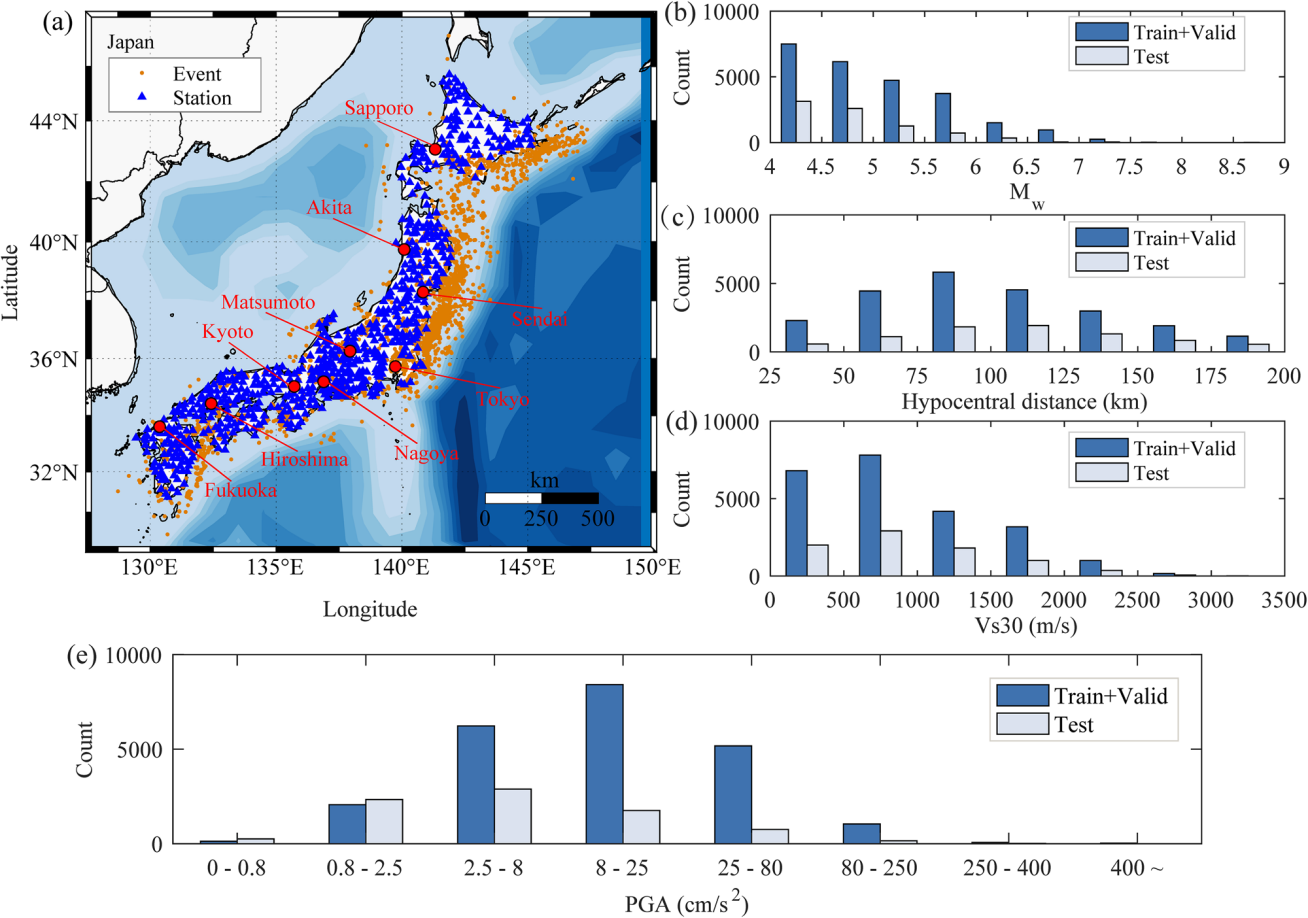
Distribution of the selected Japan accelerograms. (**a**) Distribution of accelerograms with Mw and hypocentral distance. The maps are drawn using M_map^58^. (**b**) Number of accelerograms with M_w_. (**c**) Number of accelerograms with hypocentral distance. (**d**) Number of accelerograms with Vs30. (**e**) Number of accelerograms with PGA.

Surface acceleration records from the Kiban Kyoshin Network (KiK-net) database (National Research Institute for Earth Science and Disaster Resilience, 2019)^56^ in Japan are used to establish training, validation, and testing datasets in this article. The database includes 3271 earthquake events of magnitude 4–9 recorded at 650 stations from February 10, 1998, to December 18, 2021. All earthquake events have a latitude range of 29° N–47° N and a longitude range of 128° E–148° E. Figure 1a shows the distribution of KiK-net earthquake events and stations used in this article. When filtering acceleration records, to ensure that the first arrival seismic wave has at least a 3 s P wave^53–55^ and includes offshore seismic events as much as possible, the epicentral distance is limited to 25 to 200 km. 25 km is to avoid the warning blind zone^53,64,65^, while 200 km is to include seismic events near the coast. To reduce the impact of noise, seismic records with a signal-to-noise ratio (SNR) of no less than 10 dB ^40^ were selected. Earthquake records with an SNR greater than 10 dB are common. If the SNR of a record is less than 10 dB, it is likely that there is a malfunction in the monitoring instrument or abnormal vibrations in the surrounding environment of the monitoring station. Additionally, considering the impact of site conditions on ground motion^57,59^, it is ensured that the site data of the station includes Vs30 (average shear wave velocity at 30 m underground). Next, routine processing is performed on the filtered records, including checking the baseline, unifying the sampling rate to 100 Hz, automatically picking up P-waves and manually verifying them. Finally, the peak ground acceleration (PGA) calculated by synthesizing the three-component vectors is used as the final PGA of each group of records. The synthetic method is the square root of the sum of the squares. After the above data selection and processing, a total of 31,303 sets of three-component surface acceleration records were selected.

Considering the sequence of earthquake occurrence times, 17,817 records from 1998 to 2014 were selected as the training dataset for training the DLPGA, accounting for approximately 59.65% of the total records. The 5329 records from 2015 to 2018 were used as a validation dataset to optimize the architecture and hyperparameters of DLPGA, accounting for approximately 17.02% of the total records. The 8157 records from 2019 to 2021 were used as test datasets to test the effect of the DLPGA model on predicting PGA, accounting for approximately 26.05% of the total records. This method of dividing datasets according to the time of earthquake occurrence ensures the independence of seismic events (avoiding the division of records from a single earthquake event into the 3 datasets). The distribution of the number of records in each dataset with magnitude, source distance, and Vs30 is shown in Fig. 1b–d, and e shows the statistical distribution of PGA in each dataset.

## Methodology

**Figure 2:**
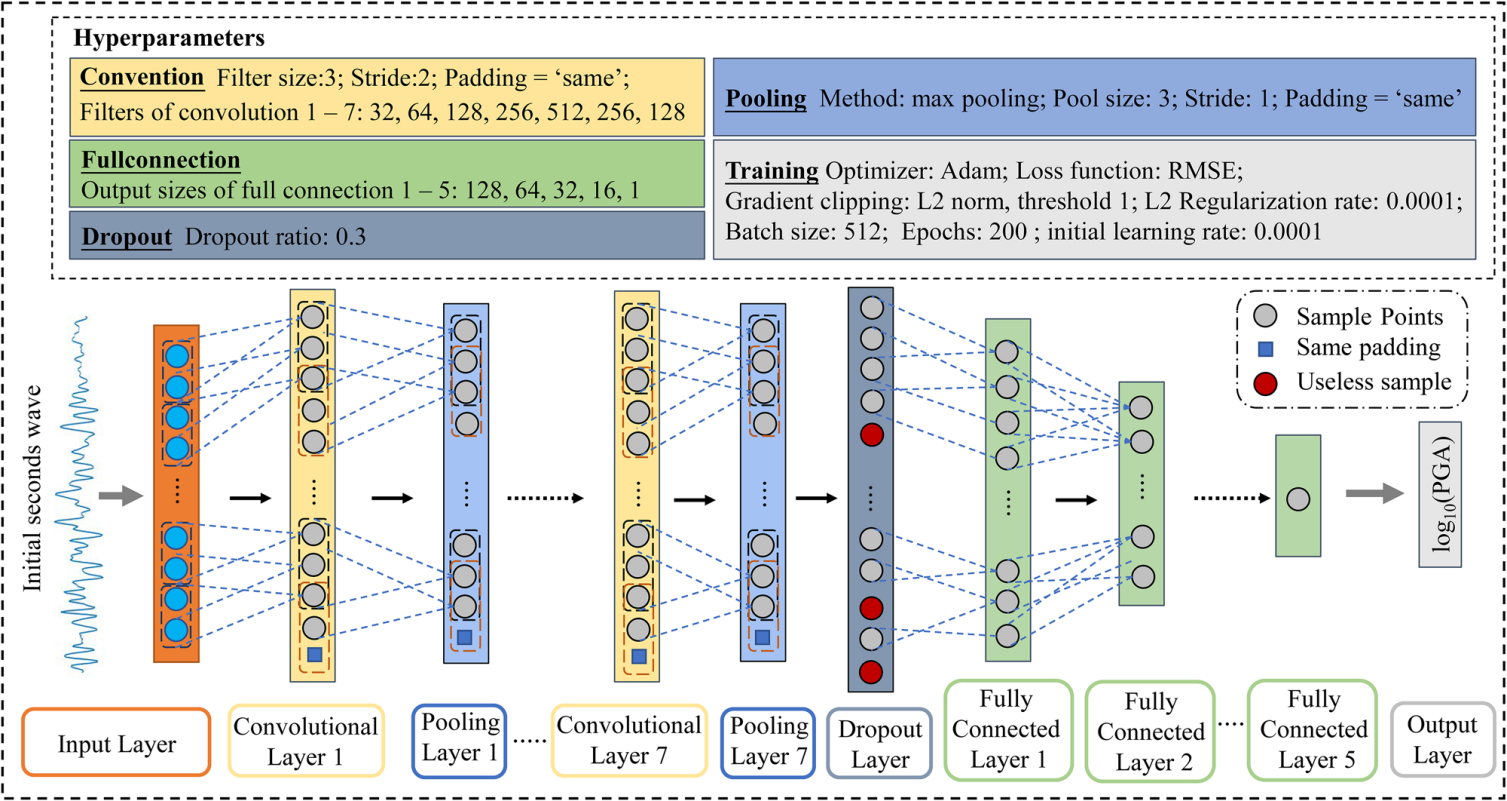
Architecture and hyperparameters of DLPGA.

In this study, we constructed DLPGA based on a one-dimensional multilayer convolutional neural network (CNN). The architecture and hyperparameters of DLPGA determine its performance. The detailed settings of the DLPGA architecture and hyperparameters in this article are shown in Fig. 2. The DLPGA was divided into four parts, including the input layer, hidden layer, fully connected layer, and output layer. First, for the input layer, the input of DLPGA is the vertical first arrival seismic wave (acceleration record) of a single station. Accelerations with different first-arrival seismic wave durations will have different data lengths, such as 300 data points when inputting an initial 3 s seismic wave. Second, the hidden layer is used to extract the most relevant features of PGA in the first arrival seismic wave. The hidden layer consists of 7 convolutional layers (kernels of size 3; stride 2; padding: same), 7 maximum pooling layers (kernels of size 3; stride 1; padding: same), and 1 dropout layer (dropout ratio: 0.3). Each convolutional layer is followed by a pooling layer, and the last pooling layer is followed by a dropout layer. The activation function of the convolution operation uses the rectified linear unit (ReLU) function to achieve nonlinearization^60^. To prevent overfitting, the convolution operation also uses L2 norm regularization with a regularization rate of 0.0001^50^. The role of the pooling layer is to downsample the features, and the dropout layer randomly discards some features to prevent overfitting^61^. Third, there are 5 fully connected layers, and the output size of each fully connected layer is shown in Fig. 2. Its function is to perform regression calculations on the extracted features. Last, for the output layer, DLPGA uses regression output to calculate log10 (PGA). During the training of the DLPGA, the Adam (adaptive moment estimation) optimizer was used for optimization training^62^. The initial learning rate is 0.0001, the number of batch samples is 512, and the root mean square error (RMSE) is used as the loss function in the training model. In the training model, RMSE is used as the loss function. When the number of training epochs reaches 200 or the loss of the validation dataset does not decrease for 10 consecutive rounds, the training is stopped.

**Figure 3:**
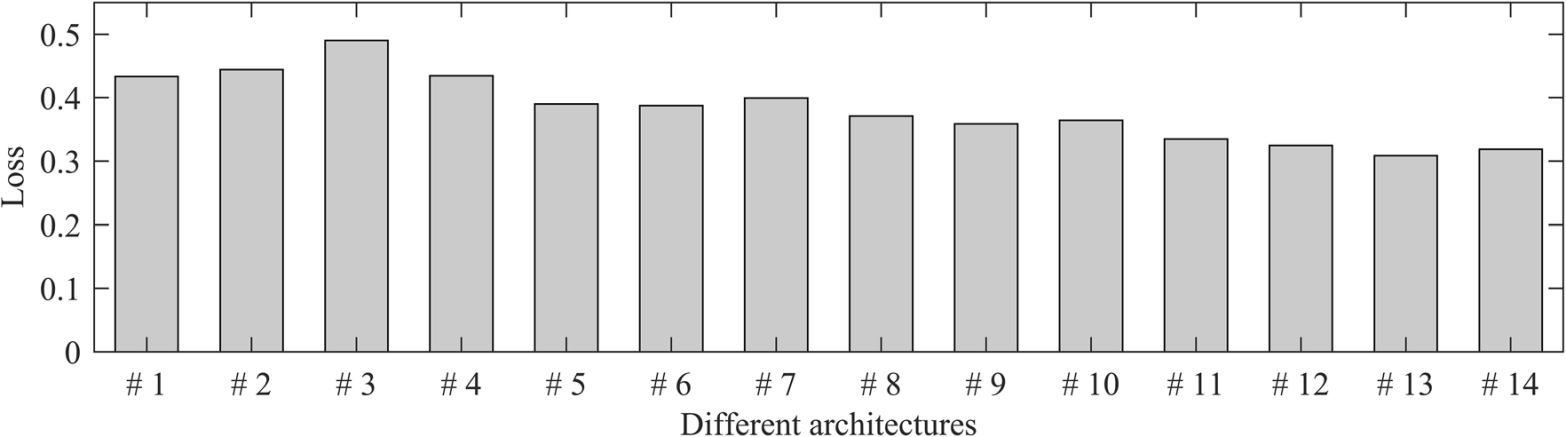
Loss of PGA predicted by different architectures with initial 3 s waves of the validation dataset.

The above DLPGA architecture and hyperparameters determine its predictive performance. However, there is no rule or principle to determine the architecture and hyperparameters, which can only be determined by repeated trial-and-error of the training dataset and validation dataset. For example, when determining the architecture of the DLPGA, after fixing the hyperparameters to some conventional settings, some architectures are set for trial calculation (Table 1, only partial architectures), and the architecture with the minimum loss value is selected from the trial results (Fig. 3). It should be noted that the architecture and hyperparameter settings have infinite possibilities, and the optimal setting can only be selected in a finite number of trial-and-error attempts.

**Table 1:** Adjustments of DLPGA architectures.

Number	Number of convolutions	Activation	Pooling method	Dropout ratio	Number of full connections
DLPGA#1	5	ReLU	Max pooling	0.1	1
DLPGA#2	5	ReLU	Average pooling	0.1	1
DLPGA#3	5	ELU^a^	Average pooling	0.1	1
DLPGA#4	5	ELU	Max pooling	0.1	1
DLPGA#5	5	ReLU	Max pooling	0.2	1
DLPGA#6	5	ReLU	Max pooling	0.3	1
DLPGA#7	5	ReLU	Max pooling	0.4	1
DLPGA#8	6	ReLU	Max pooling	0.3	1
DLPGA#9	7	ReLU	Max pooling	0.3	1
DLPGA#10	8	ReLU	Max pooling	0.3	2
DLPGA#11	7	ReLU	Max pooling	0.3	3
DLPGA#12	7	ReLU	Max pooling	0.3	4
DLPGA#13	7	ReLU	Max pooling	0.3	5
DLPGA#14	7	ReLU	Max pooling	0.3	6

^a^ELU is an exponential linear unit^63^.

### Test

To balance the timeliness and accuracy of early warning information, the EEW system generally starts calculating and publishing early warning information when the station monitors the first-arrival 3 s P wave^53,64–68^. In addition, during the warning process, the EEW system will continuously update the warning information with the increase in the first-arrival seismic waves to ensure the accuracy of the warning information. Therefore, we use the test dataset to first test the effectiveness of DLPGA in predicting PGA with the initial 3 s P wave and then increase the duration of the first-arrival seismic wave from 3 to 6 s to test the effectiveness of DLPGA in continuously predicting PGA. The prediction effectiveness of the DLPGA is evaluated by comparison with the current commonly adopted Pd method^38^. The empirical formula for the prediction of PGA by the Pd method is shown in Eq. (1):

1 $${log}_{10}\left({\text{PGA}}\right)=a\times {log}_{10}\left({\text{Pd}}\right)+b$$

where a and b are the fitting coefficients and Pd is the displacement amplitude of the first-arrival P wave. Pd is obtained through twice integration calculations, and after each integration, a 0.075 Hz high-pass Butterworth filter is commonly used to avoid drift caused by low-frequency noise^55,64,66^. For the first-arrival 3–6 s seismic waves, the fitting coefficients a and b in empirical Eq. (1) were determined from the training dataset and validation dataset, as shown in Table 2.

**Table 2:** Fitting coefficients with initial 3–6 s waves.

Time (s)	*a*	*b*
3	0.6874	2.5649
4	0.7265	2.7684
5	0.7591	3.0853
6	0.7923	3.2985

### Prediction results of DLPGA with initial 3 s waves

**Figure 4:**
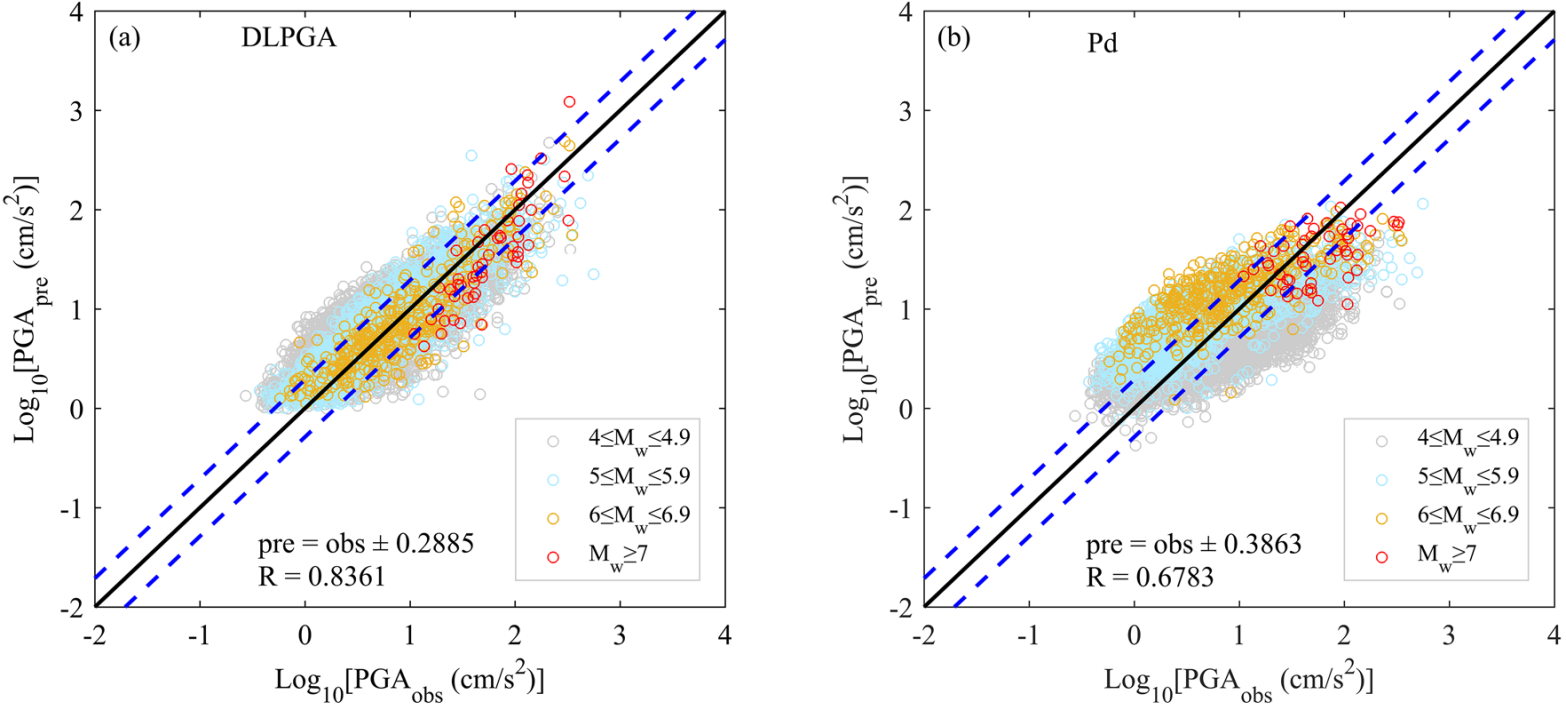
The distribution of PGAs predicted by the DLPGA (**a**) and Pd (**b**) with the initial 3 s waves of the test dataset. Different colored circles represent different magnitudes of PGA prediction. The black solid line is the 1:1 line showing perfect agreement between the predicted and observed values. The two blue dashed lines represent the range of ± 1 standard deviation. R is the correlation coefficient. pre is the predicted PGA (PGA_pre_), and obs is the observed PGA (PGA_obs_).

Figure 4 shows the distribution of PGA predicted by DLPGA and Pd with the initial 3 s waves of the test dataset. According to the distribution of the predicted PGA, the PGA predicted by the DLPGA shows a good linear distribution with the actual PGA (observations), which is more uniformly distributed on both sides of the 1:1 line. The PGA predicted by Pd is generally more discrete and tends to be larger (overestimation of small values) when the actual PGA is small and smaller (underestimation of large values) when the actual PGA is large. Especially for earthquakes with magnitudes of 6 to 6.9, the PGA predicted by Pd is generally larger than the actual value. In addition, the correlation coefficient between the PGA predicted by DLPGA and the actual PGA increased by 23% compared to Pd, and the error standard deviation of the PGA predicted by DLPGA decreased by approximately 25% compared to the Pd method.

**Figure 5:**
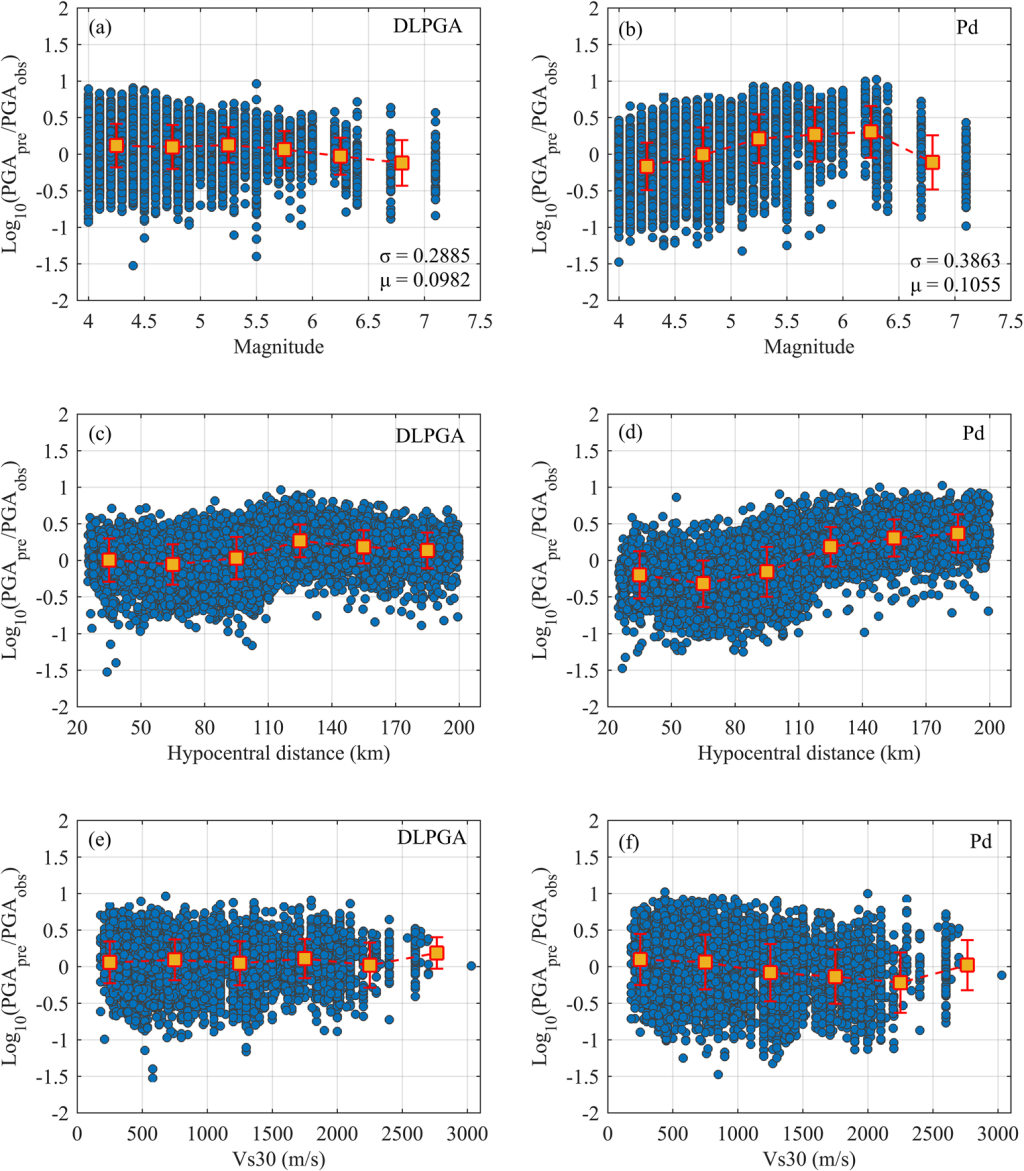
Distributions of DLPGA and Pd prediction error with magnitude (**a**,**b**), epicentral distance (**c**,**d**) and Vs30 (**e**,**f**) with initial 3 s waves of the test dataset. Blue circles represent error values, yellow squares represent the mean error value and red error lines indicate the standard deviation of the error.

To further analyze the influencing factors of the two methods for predicting PGA, the distribution of prediction errors with magnitude, source distance and Vs30 and their error bar variations are plotted, as shown in Fig. 5. The magnitude is divided into 5 sections according to the interval of 0.5 magnitude units from magnitude 4 to 7.5. The epicentral distance is divided into 5 sections from 20 to 200 km at intervals of 30 km. Vs30 is divided into 5 sections from 0 to 3000 m/s at intervals of 500 m/s. The yellow square in the error bar represents the error mean of each section, reflecting the overall trend of the error. The length of the two short columns above and below is the standard deviation of the error for each section, reflecting the degree of dispersion of the error with changes in magnitude, source distance, and Vs30. From the distribution of errors in predicting PGA with magnitude changes and the variation in error bars (Fig. 5a,b), it can be seen that the prediction errors of the DLPGA are evenly distributed with magnitude changes, and the overall trend is relatively flat. After the magnitude is greater than 6, the error tends to gradually decrease, the position of the error bars is near 0 error, and the trend of change is relatively flat. The overall dispersion of the prediction errors of Pd is large and unevenly distributed, with a large overall variation. For magnitudes 4–5.5, the error bars are increasing, and for magnitudes greater than 6, the error bars are decreasing. The error standard deviation of the DLPGA in each magnitude band is smaller than that of Pd. From the distribution of the errors with the epicentral distance (Fig. 5c,d), it can be seen that when the epicentral distance is greater than approximately 110 km, the errors of DLPGA have a slightly larger trend, but the overall dispersion is smaller. The error of Pd tends to be significantly larger when the epicentral distance is greater than approximately 110 km, and the degree of dispersion is greater than that of DLPGA. Moreover, within each epicentral distance range, the error standard deviation of DLPGA in predicting PGA is smaller than that of Pd. From the distribution of error with Vs30 (Fig. 5e,f), it can be seen that the distribution of DLPGA prediction errors is more centrally distributed near the value of 0, and the overall trend of change does not show significant fluctuations. The prediction errors of Pd have a tendency to be smaller when Vs30 is larger than approximately 1000 m/s, and the distribution of errors is more discrete. The standard deviation of the prediction errors of the DLPGA is smaller than that of Pd within each Vs30 band.

### Prediction results of DLPGA with initial 4–6 s waves of the test dataset

**Figure 6:**
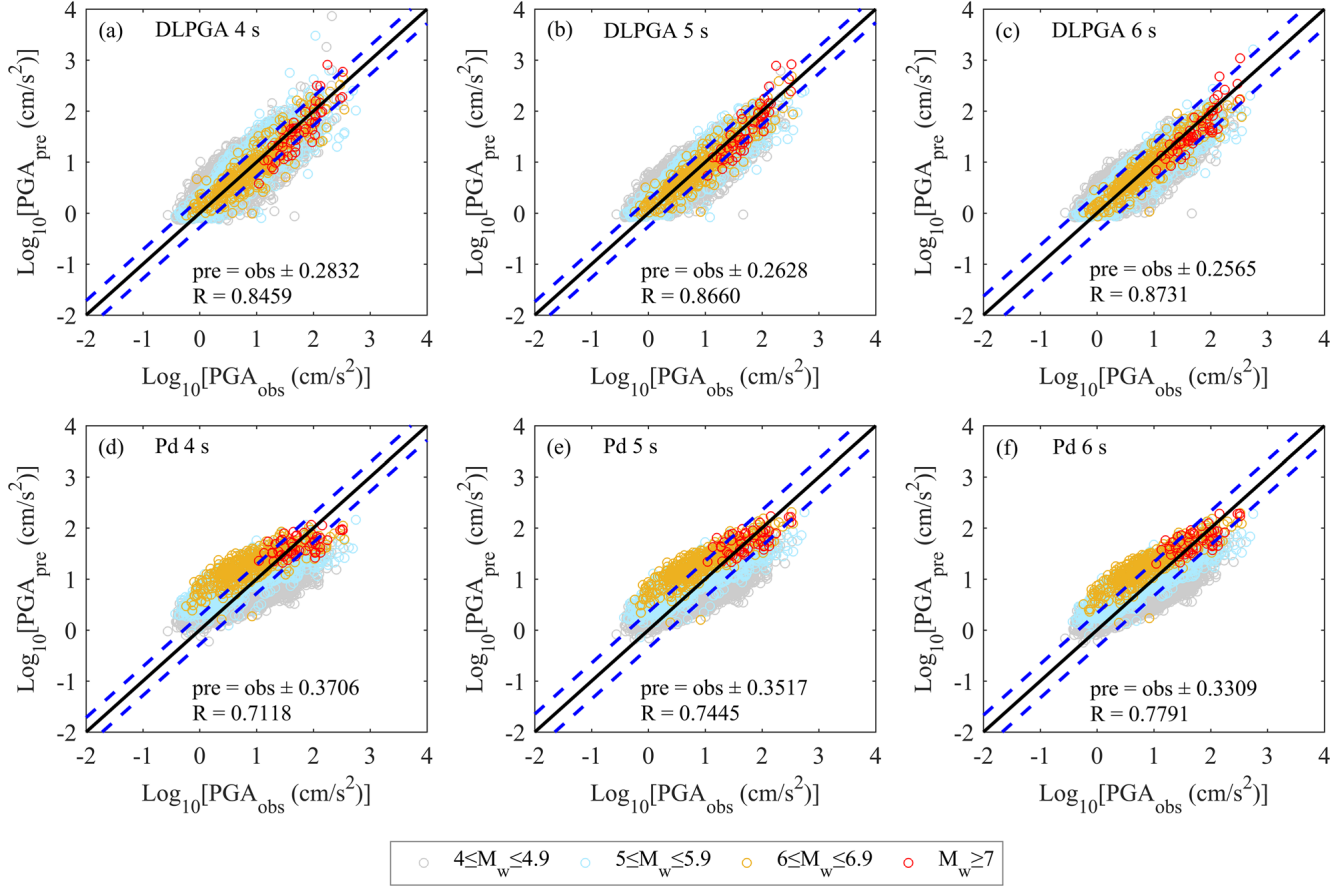
The distribution of PGA predicted by DLPGA with initial 4–6 s waves of the test dataset. Different colored circles represent different magnitudes of PGA prediction. The black solid line is the 1:1 line showing perfect agreement between the predicted and observed values. The two blue dashed lines represent the range of ± 1 standard deviation. R is the correlation coefficient. pre is the predicted PGA (PGA_pre_), and obs is the observed PGA (PGA_obs_).

Figure 6 shows the linear relationship between the PGA predicted by DLPGA and Pd and the actual PGA with initial 4–6 s waves in the test dataset. From the distribution of the predicted PGA, as the initial wave increases from 4 to 6 s, the PGA predicted by the DLPGA can be more concentrated on both sides of the 1:1 line. The PGA predicted by Pd is constantly improving, but the phenomenon of “small value overestimation” and “large value underestimation” has not been significantly improved.

**Figure 7:**
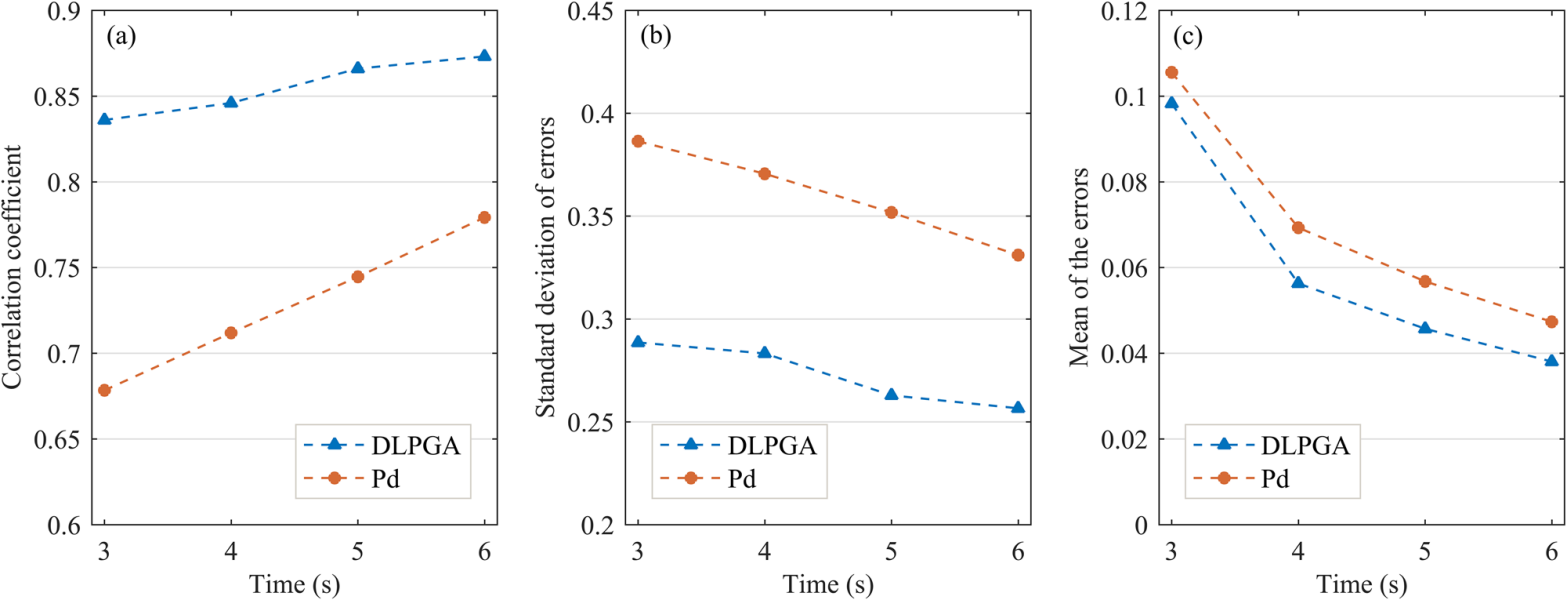
Correlation coefficient (**a**), standard deviation of error (**b**) and mean of the errors (**c**) of PGA predicted by DLPGA and Pd with initial 3–6 s waves of the test dataset.

Figure 7 further demonstrates the variation in the correlation coefficients, error standard deviation and error mean of the prediction results of the DLPGA and Pd with the increase in the duration of the first-arrival seismic wave. From the correlation coefficients between the predicted PGA and the actual PGA (Fig. 7a), it can be seen that the correlation coefficients obtained by the two methods increase with the increase in the duration of the first-arrival seismic wave, the correlation coefficient of the DLPGA increases from 0.8361 to 0.8731, the correlation coefficient of Pd increases from 0.6783 to 0.7791, and the correlation coefficient of the DLPGA is always larger than that of Pd and is approximately 12–23% higher than that of Pd. In the results of the error standard deviation (Fig. 7b), the error standard deviation of the PGA predicted by both methods decreases with the increase in the duration of the first-arrival seismic wave, the error standard deviation of the DLPGA decreases from 0.2885 to 0.2565, that of Pd decreases from 0.3863 to 0.3309, and that of the DLPGA is consistently smaller than that of Pd and approximately 22–25% lower than that of Pd. In the results of the error mean (Fig. 7c), the error mean of the PGA predicted by the two methods is close to zero with the increase in the duration of the first-arrival seismic wave, the error mean of the DLPGA decreases from 0.0982 to 0.0380, that of Pd decreases from 0.1055 to 0.0473, and the error mean of the DLPGA is always smaller than that of Pd and approximately 6.92–19.66% lower than that of Pd. Notably, combining the results of Figs. 4, 6 and 7, it can be seen that the accuracy of the DLPGA in predicting the PGA with an initial 3 s wave is already better than that of Pd with an initial 6 s wave.

## Generalization test

### Generalization dataset

**Figure 8:**
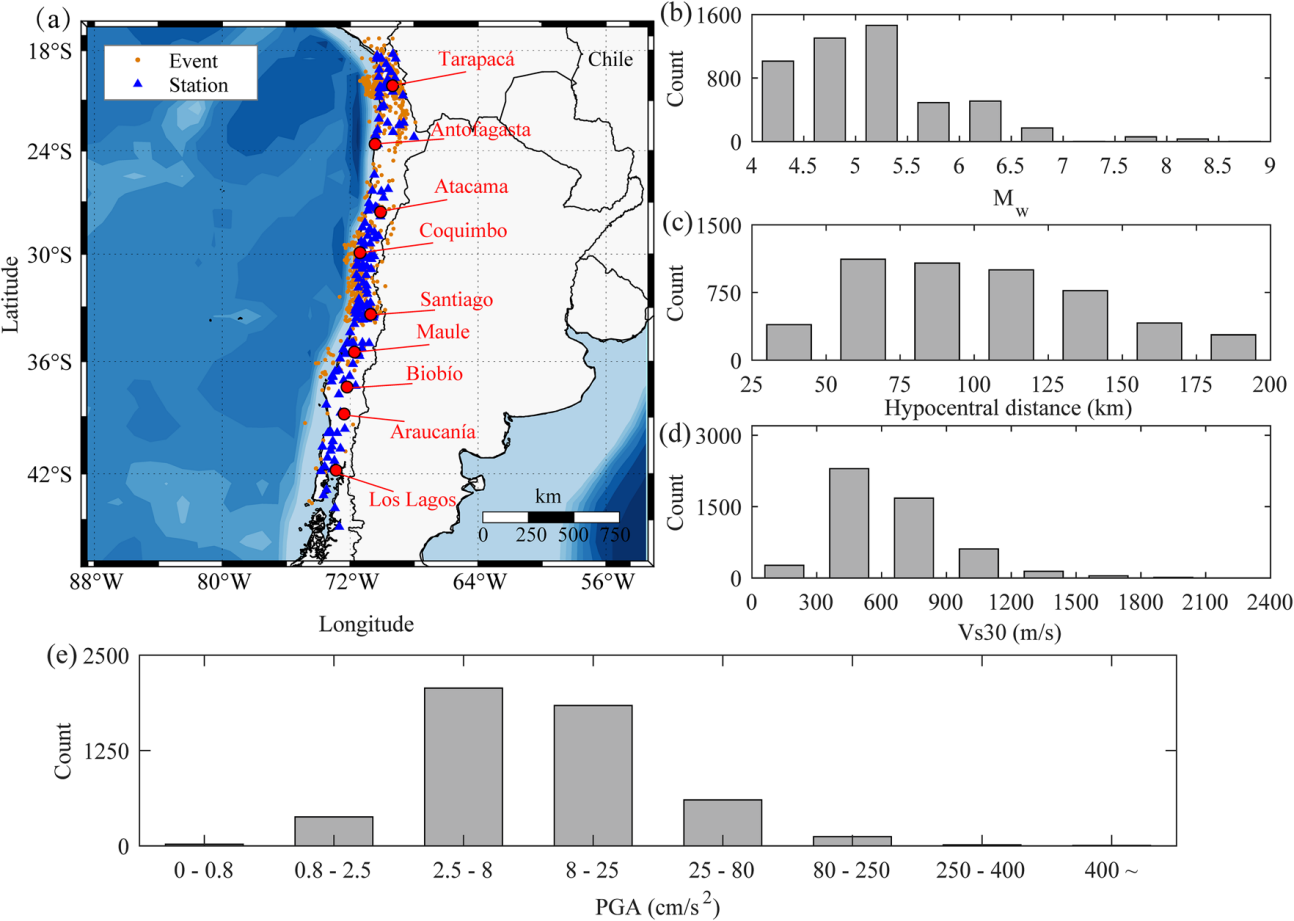
Distribution of the selected Chile accelerograms. (**a**) Distribution of accelerograms with Mw and epicentral distance. The maps are drawn using M_map^58^. (**b**) Number of accelerograms with M_w_. (**c**) Number of accelerograms with epicentral distance. (**d**) Number of accelerograms with Vs30. (**e**) Number of accelerograms with PGA.

In the aforementioned tests, Japanese earthquake records are used in the training dataset, validation dataset, and test dataset. All three datasets contain some similar regional information, such as similar seismic sources, propagation paths, site conditions, and monitoring instruments, making it difficult to assess the prediction effectiveness of the DLPGA in non-Japanese regions. To test the effectiveness of DLPGA trained with Japanese earthquake records in other regions, the generalization ability of DLPGA was tested using Chilean earthquake records. Generalization ability refers to the ability of machine learning algorithms to adapt to new samples. A total of 5053 sets of three-component acceleration records were screened from the Chilean SIBER-RISK database using the same screening and processing methods as the Japanese records. A total of 1617 seismic events of magnitude 4–9 recorded at 229 stations from 3 March 1985 to 21 July 2021 were included, with latitudes ranging from 18° to 42° S and longitudes from 67° to 74° W. A generalization dataset was created based on strong motion data from Chile. The generalization dataset was not involved in training the DLPGA and was not used to fit empirical formulas of Pd versus PGA. The distribution of seismic events and stations in the generalization dataset, as well as the magnitude, epicentral distance, Vs30 and PGA, are shown in Fig. 8.

### Prediction results of DLPGA with initial 3–6 s waves

**Figure 9:**
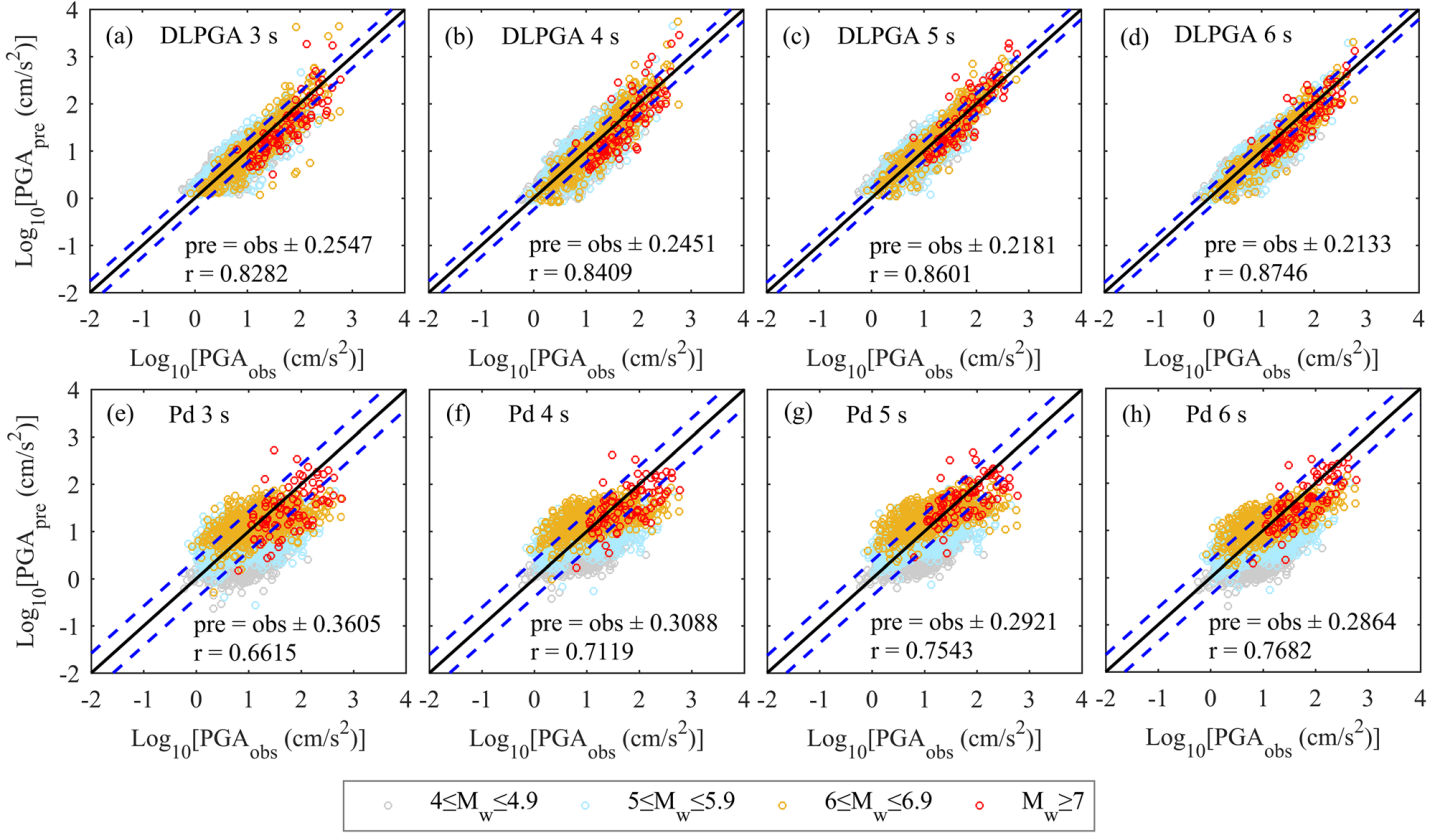
The distribution of PGA predicted by DLPGA and Pd with initial 3–6 s waves of the generalization dataset. Different colored circles represent different magnitudes of PGA prediction. The black solid line is the 1:1 line showing perfect agreement between the predicted and observed values. The two blue dashed lines represent the range of ± 1 standard deviation. R is the correlation coefficient. pre is the predicted PGA (PGA_pre_), and obs is the observed PGA (PGA_obs_).

The linear relationship between the PGA predicted by the DLPGA and Pd and the actual PGA is shown in Fig. 9 when the initial 3–6 s vertical seismic waves are taken as inputs from the generalization dataset. From the distribution of the predicted PGA, the PGA predicted by the DLPGA is uniformly and centrally distributed on both sides of the 1:1 line, and the PGA predicted for all magnitude ranges has similar distributions. The dispersion of the PGA predicted by Pd is large, and the phenomena of "overestimation of small values" and "underestimation of large values" are obvious. The PGA predicted by Pd is large for earthquakes of magnitude 6–6.9. Generally, with the increase in the duration of the first-arrival seismic wave, the effect of the two methods in predicting the PGA is continuously improved, and the DLPGA is always significantly better than Pd.

**Figure 10:**
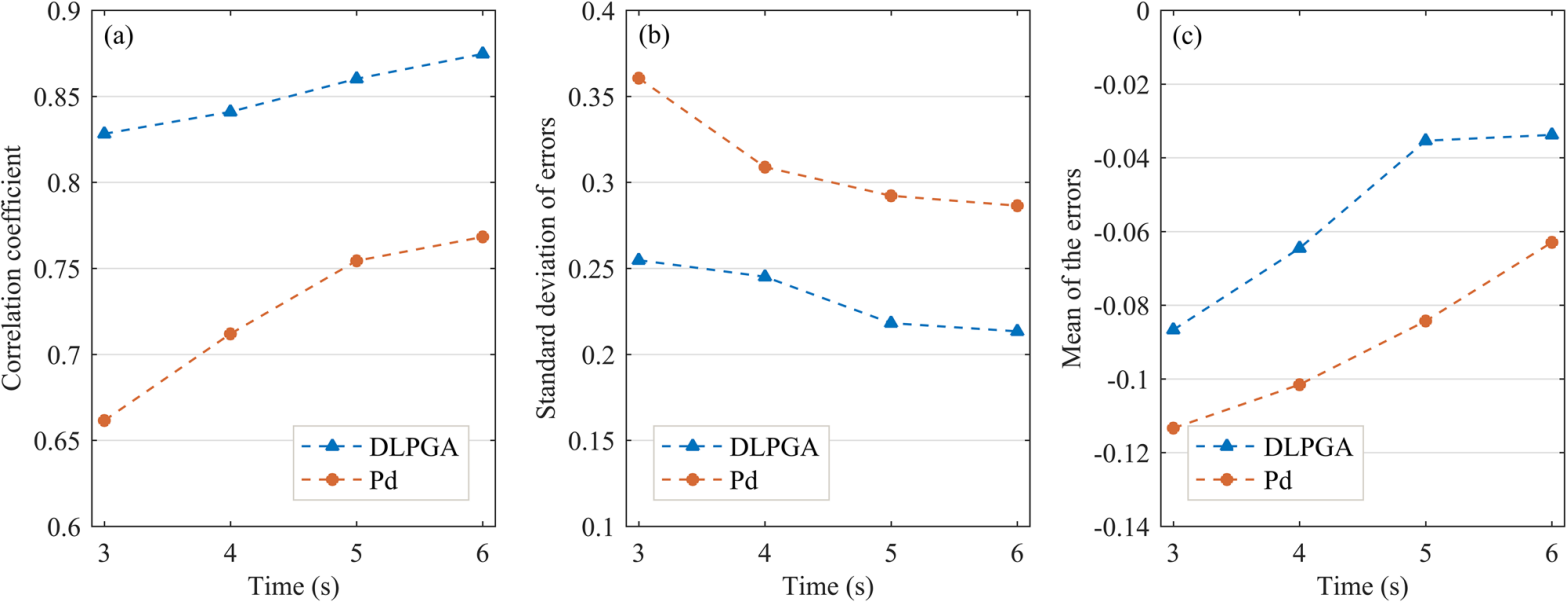
Correlation coefficient (**a**), standard deviation of error (**b**) and mean of the errors (**c**) of PGA predicted by the DLPGA and Pd with the initial 3–6 s waves of the generalization dataset.

Figure 10 further illustrates the variation in the correlation coefficients, standard deviation of error and error mean of PGA predicted by DLPGA and Pd with increasing first-arrival seismic wave duration. From the results of the correlation coefficients between the predicted PGA and the actual PGA (Fig. 10a), it can be seen that the correlation coefficients obtained by both methods increase with the increase in the first-arrival seismic wave duration. The correlation coefficient of DLPGA increases from 0.8282 to 0.8746 and that of Pd increases from 0.6615 to 0.7682. The correlation coefficient of DLPGA is always greater than that of Pd and is approximately 15.60–25.20% higher than that of Pd. From the results of the error standard deviation (Fig. 10b), it can be seen that the error standard deviation of the PGA predicted by both methods decreases with increasing first-arrival seismic wave duration. The error standard deviation of the DLPGA decreases from 0.2547 to 0.2133 and that of Pd decreases from 0.3605 to 0.2864. The error standard deviation of the DLPGA is always smaller than that of Pd and is approximately 25.52–29.35% lower than that of Pd. Combining the results of Fig. 9 and 10, it can be seen that the accuracy of DLPGA in predicting PGA with initial 3 s waves is already better than that of Pd with initial 6 s waves.

Comparing the generalization test results of the Chilean data (Figs. 9 and 10) and those of the Japanese data (Figs. 4, 6 and 7), it can be seen that the distribution, correlation coefficient, error standard deviation and error mean of the PGA predicted by the DLPGA are relatively close in both datasets, which can be continuously improved with increasing seismic wave duration and are always far better than those of Pd.

### Discrimination of destructive ground motion

In the practical application of EEW, the threshold of ground motion parameters is mainly used in EEW systems to determine whether the ground motion is destructive^2,12,69,70^. Therefore, it is necessary to evaluate the effectiveness of the DLPGA in discriminating destructive earthquakes for the practical application of EEW. In this paper, 25 cm/s^2^ is used as the PGA threshold to distinguish seismic destructiveness. Because PGA = 25 cm/s^2^ is equivalent to Modified Mercalli Intensity (MMI) IV^14^, the earthquake waves have a slight destructive effect, requiring the issuance of warning messages^14,51,71^, In the generalization dataset, there are 4310 nondestructive earthquake records and 743 destructive earthquake records. Using thresholds to discriminate the destructiveness of earthquakes is a binary classification problem. Therefore, a confusion matrix can be used to evaluate the destructiveness of earthquakes. The four basic evaluation indexes of the confusion matrix are defined as follows:

- True Positive (TP): When the actual log10(PGA) is less than log10(25), the predicted log10(PGA) is less than log10(25);
- True Negative (TN): When the actual log10(PGA) is greater than or equal to log10(25), the predicted log10(PGA) is greater than or equal to log10(25);
- False Positive (FP): When the actual log10(PGA) is less than log10(25), the predicted log10(PGA) is greater than or equal to log10(25);
- False Negative (FN): When the actual log10(PGA) is greater than or equal to log10(25), the predicted log10(PGA) is less than log10(25).

**Figure 11:**
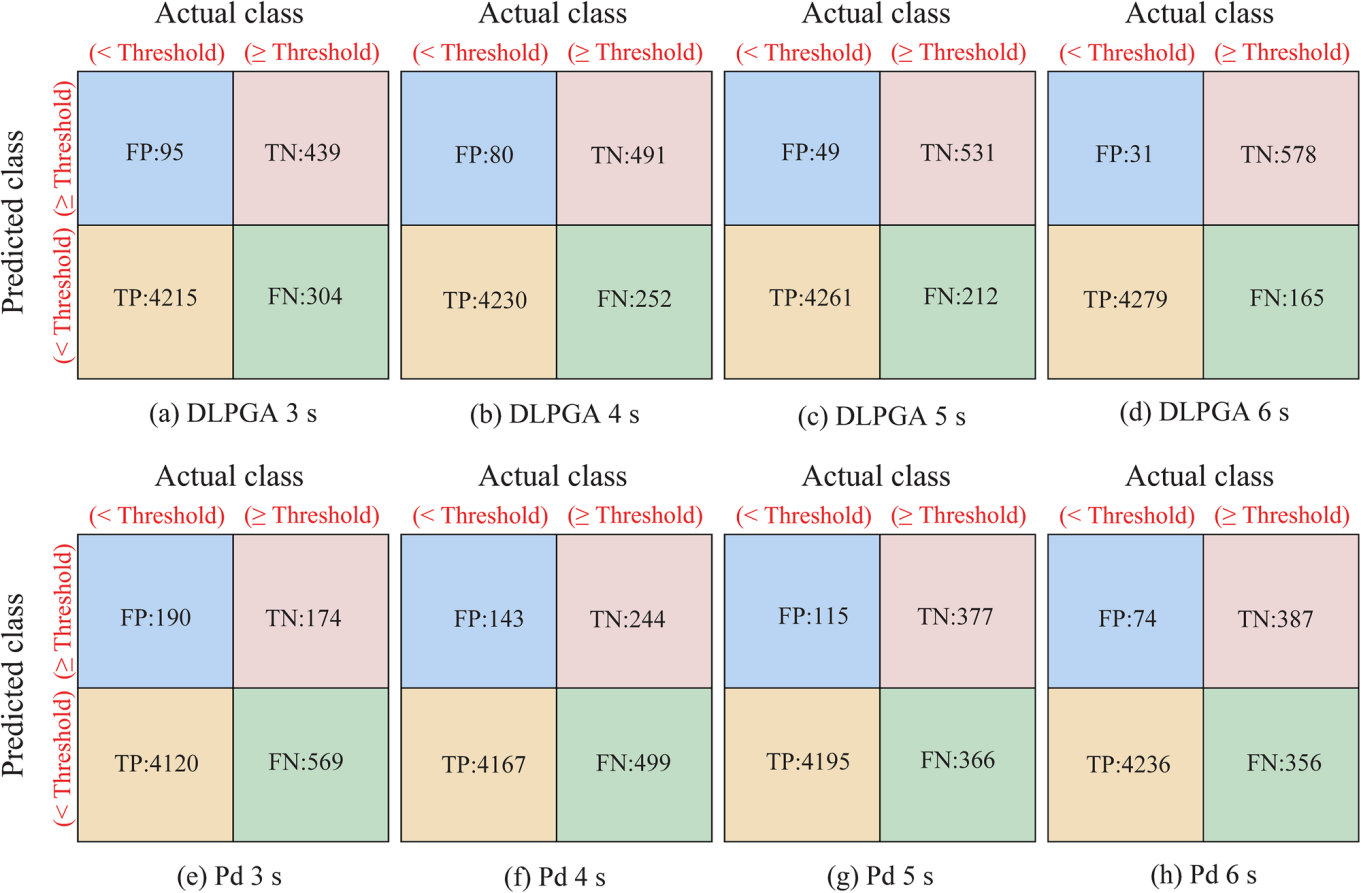
Confusion matrices for PGA threshold for generalization dataset.

Figure 11 shows the confusion matrices of the DLPGA and Pd methods with seismic waves of different initial lengths in the generalization dataset. In general, the TP and TN of the DLPGA are always larger than those of Pd, while the FP and FN of the DLPGA are always smaller than those of Pd, which indicates that the prediction effectiveness of the DLPGA is better than that of Pd. It should be noted that more records are determined as FN in both DLPGA and Pd, which, together with Fig. 9, is mainly due to the problem of "large value underestimation".

To integrate the four indicators of the confusion matrix (FP, TN, TP, and FN) for a more intuitive comparison, the Matthews correlation coefficient (MCC) was calculated^72^. The MCC is a correlation coefficient that describes the relationship between actual classification and predicted classification, taking into account the four basic evaluation indicators in the confusion matrix. The value range of MCC is [− 1,1], and the higher the value of MCC is, the higher the discrimination accuracy, as calculated by formula (2).

2 $${\text{MCC}}=\frac{{\text{TP}}\times {\text{TN}}-{\text{FP}}\times {\text{FN}}}{\sqrt{\left({\text{TP}}+{\text{FP}}\right)\times \left({\text{TP}}+{\text{FN}}\right)\times \left({\text{TN}}+{\text{FP}}\right)\times \left({\text{TN}}+{\text{FN}}\right)}}$$

**Figure 12:**
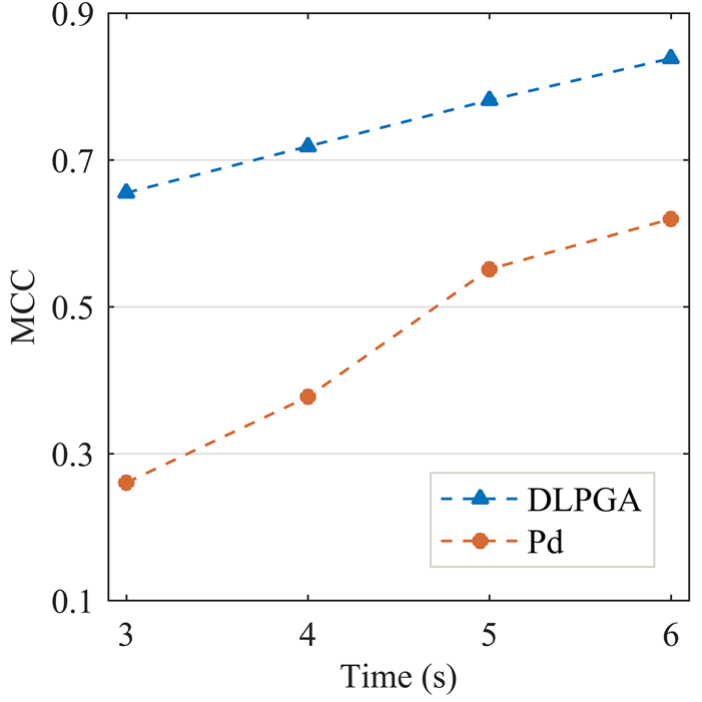
MCC of DLPGA and Pd with initial 3–6 s waves.

Figure 12 gives the variation in MCC with the duration of the first-arrival seismic wave of the DLPGA and Pd methods. From the figure, it can be seen that the MCC of the DLPGA is much better than that of Pd; when the duration of the first arrival seismic wave increases from 3 to 6 s, the MCC of the DLPGA increases from 0.6552 to 0.8384, and the MCC of Pd increases from 0.2609 to 0.6195. The MCC of DLPGA is 35–150% higher than that of Pd. The MCC of the DLPGA with the initial 3 s wave is larger than that of Pd with the initial 6 s. The results of the MCC indicate that the DLPGA can more quickly identify the destructiveness of ground motion than Pd, and the accuracy is improved by 35–150%.

## Discussion

In this study, a deep learning model (DLPGA) based on a multilayer CNN is proposed to improve the accuracy of an on-site EEW system in predicting PGA. The training, verification and testing of DLPGA are completed by using the surface acceleration records of the KiK-net database in Japan. In the test results of the initial 3–6 s waves, the error standard deviation and error mean of the DLPGA in predicting PGA are always smaller than those of Pd, and the correlation coefficient is always larger than that of Pd. Moreover, the accuracy of DLPGA in predicting PGA with initial 3 s waves is better than that of Pd with initial 6 s waves, indicating that PGA is predicted by DLPGA faster and more accurately than Pd. In addition, the accuracy of DLPGA in predicting PGA is less affected by magnitude, epicentre distance, and Vs30 than Pd, indicating that DLPGA has better applicability than Pd. To further evaluate the effectiveness of DLPGA trained with Japanese data in predicting PGA in other regions, a generalization test was conducted using Chilean surface acceleration records. In the results of the generalization test for the initial 3–6 s, the distribution, correlation coefficient, standard deviation of error, and error mean of the PGA predicted by the DLPGA are close to the results of the Japanese data and are much better than those of Pd, which indicates that the DLPGA has a better generalization ability than Pd. The accuracy of the DLPGA in determining the destructiveness of ground motion is significantly higher than that of Pd. The DLPGA is more accurate with an initial 3 s seismic wave than Pd with an initial 6 s seismic wave, which indicates that the DLPGA is faster and more accurate in determining the destructiveness of earthquakes than Pd. The DLPGA is significantly better than Pd in terms of accuracy and timeliness. The main reason is that the DLPGA avoids human empirical interference by automatically extracting information related to the PGA and maximizes the retention of important information related to the PGA in the initial wave. Pd is a feature parameter based on empirical definitions. The larger the PGA is, the larger the surface deformation is, and Pd can only represent a certain aspect of the information related to PGA in the initial wave.

Compared with the PGA prediction method proposed by Jozinovi 'c et al.^50^, Hsu et al.^51^ and Chiang et al.^52^, DLPGA has the following four advantages in the advancement of the algorithm: (1) DLPGA uses the initial 3–6 s waves as inputs, while the CNN model of Jozinovi´ c et al. requires at least initial 10 s waves as inputs; thus, DLPGA has better timeliness. (2) The DLPGA only uses initial vertical seismic waves, while Jozinovi 'c et al.^50^, Hsu et al.^51^ and Chiang et al.^52^ all use three-component initial seismic waves. The initial vertical seismic waves have a better signal-to-noise ratio than the two horizontal seismic waves and have more advantages in data quality. (3) DLPGA does not preprocess the input data, whereas Hsu et al. preprocess the input data in both the time domain and the frequency domain, which increases the algorithm implementation difficulty as well as the risk of input data distortion. (4) DLPGA directly outputs PGA in the form of regression calculations, and Chiang et al.^52^ are only able to predict whether the PGA exceeded a preset threshold.

Although the DLPGA shows good prediction results, there are still some problems with the DLPGA, which need to be improved in subsequent studies. First, the PGA predicted by DLPGA has a certain degree of "underestimation of large values", which can lead to the identification of destructive earthquakes as nondestructive earthquakes. Although increasing the duration of the initial wave can improve the underestimation of large values, it will reduce the timeliness of EEW. Second, the number of seismic records with larger PGAs in the training dataset is much less than that with smaller PGAs, and the impact of data imbalance on prediction performance is unknown. Furthermore, in addition to the influence of magnitude, distance and Vs30 on the DLPGA prediction results, more complex source and site factors, such as fault rupture, soil layer and topography of the site, need to be further studied. Finally, DLPGA is implemented based on a standard CNN model, and it is necessary to optimize DLPGA in terms of the model design by adopting other strategies (e.g., the attention mechanism) to improve the prediction accuracy of PGA.

## Conclusions

We propose a PGA prediction method, DLPGA, based on CNN in deep learning. DLPGA realizes end-to-end prediction of PGA using vertical initial seismic waves as input and PGA as output. DLPGA avoids the subjectivity and one-sidedness of human-selected feature parameters by automatically extracting features from initial seismic waves. The effectiveness of DLPGA in predicting PGA is tested using Japanese seismic records, and the generalization ability of DLPGA is tested using Chilean seismic records. For initial 3–6 s seismic waves, DLPGA predicts PGA with better accuracy and timeliness than the widely used Pd. The DLPGA has a better generalization ability than Pd, and the DLPGA is able to discriminate ground motion damage faster and more accurately. This indicates that the DLPGA can replace Pd to predict PGA for on-site EEW, achieving more reliable discrimination of ground motion destructiveness.

## Data Availability

The data that support the findings of this study are available from NIED K-NET, KiK-net, National Research Institute for Earth Science and Disaster Resilience but restrictions apply to the availability of these data, which were used under license for the current study, and so are not publicly available. Data are however available from the authors upon reasonable request and with permission of NIED K-NET, KiK-net, National Research Institute for Earth Science and Disaster Resilience.
